# Assessment of sexual functions in partners of women with complaints of urinary incontinence

**DOI:** 10.1590/S1677-5538.IBJU.2015.0353

**Published:** 2016

**Authors:** Muzaffer Oguz Keles, Selahattin Caliskan, Ali Murat Gokce, Mustafa Gunes

**Affiliations:** 1 Department of Urology, Sungurlu State Hospital, Sungurlu, Corum, Turkey;; 2 Haydarpasa Numune Training and Research Hospital, Istanbul, Turkey;; 3 Department of Urology Haydarpasa Numune Training and Research Hospital, Istanbul, Turkey;; 4 Department of Urology, Faculty of Medicine, Süleyman Demirel University, Isparta, Turkey

**Keywords:** Sexual Partners, Urinary Incontinence, Erectile Dysfunction

## Abstract

**Aim:**

Investigation of the erectile functions in partners of women with urinary incontinence problems.

**Materials and Methods:**

Sexually active female patients over the age of 18 years with complaints of urinary incontinence (n=30) (Group-1), and without urinary incontinence (n=30) (Group-2, controls) were included this study. Evaluation of the patients were done at Erzincan Mengücek Gazi Training and Research Hospital's urology outpatient clinic between June 2012 and January 2013. Partners of group-1 and group-2 were asked to fill in the 5-item International Index of Erectile Function (IIEF-5) questionnaire, and then the scores of the two groups were compared for statistically significant differences.

**Results:**

Among the partners of the group-1 patients, 15 (50%) had mild erectile dysfunction (ED), 11 (36.6%) had moderate ED, 1 (3.4%) had severe ED, and erectile function was normal in the remaining 3 (10%). Among the partners of group-2 patients, 15 (50%) had mild ED, 7 had moderate ED, 1 (3.4%) had severe ED, and 7 (23.3%) had normal erectile function. Compared to the spouses of group-2 patients, ED was more prevalent among the spouses of group-1 patients.

**Conclusion:**

Erectile function in the partners of women with urinary incontinence may be adversely affected by the UI of their partners.

## INTRODUCTION

Urinary incontinence (UI), described by the International Urinary Incontinence Society as an objectively demonstrable involuntary incontinence that gives rise to social and hygienic problems, is a prevalent health problem leading to medical and psychosocial issues (1). It affects in excess of 200 million people globally, with a higher prevalence of 15-52% in females (2). Apart from causing a sense of embarrassment, lowering of self-confidence and reduced social activity, UI adversely affects the female sexual functions (3). Erectile dysfunction (ED) in men has been described as a persistent inability to achieve and maintain adequate erection to allow satisfactory sexual intercourse (4). ED has a prevalence of 5-20% in the male population, and adversely affects the life quality of the patient, his partner and/or family by giving rise to medical and psychosocial health problems (5). Although there have been many studies investigating urinary incontinence and female sexual functions (6, 7), only a few studies have reported the sexual functions of the partners of women with urinary incontinence problems (8, 9). In this study we have aimed to investigate the erectile function of the partners of women with complaints of urinary incontinence.

## MATERIALS AND METHODS

### Patient selection

Sexually active female patients referred to the Erzincan Mengücek Gazi Teaching and Research Hospital's Urology Outpatient Clinic for the first time between June 2012 and January 2013, with urinary incontinence (n=75) as the main symptom, were submitted to a preliminary investigation. Patients selected for this study were diagnosed as either stress urinary incontinence (SUI) resulting from physical activity such as coughing, sneezing, straining that causes increased intra-abdominal pressure on the bladder, or urge urinary incontinence (UUI), which is the unintentional leaking of urine caused by contraction of the bladder muscle, usually associated with a sense of urgency. Persons afflicted with both types of incontinence are diagnosed as mixed type urinary incontinence (MUI). Patients with pelvic organ prolapse (POP) were excluded from the study. The 75 female patients enrolled in the study were asked to have their partners fill in the validated 5-item International Index of Erectile Function (IIEF-5) questionnaire. Only 30 women could comply with this. Hence the partners of 30 sexually active female patients without any complaints of urinary incontinence, who completed the IIEF-5 questionnaire were included in the study as the control-group.

Demographic characteristics of the patients in both study groups, including age, partners' age, body mass index (BMI), and number of births was recorded. In the Group-1 patients with incontinence the type and duration of incontinence, the number of incontinence pads used per day, and the types and number of deliveries were recorded. The female patients in the incontinence group were also asked to complete the Urogenital Distress Inventory-Short Form UDI-6 and the Incontinence Impact Questionnaire-Short Form IIQ-7.

The criteria for exclusion from the study were defined as the reluctance of the patient to join the study, previous medical or surgical interventions for erectile dysfunction (ED), diabetes or hypertension, and a history of pelvic surgery. Written consents were obtained from those who accepted to participate and all participants were asked to complete the IIEF-5 questionnaire. ED assessment was based on IIEF-5 scores, 22-25 indicating normal erectile function, 17-21 mild ED, 12-16 mild-to-moderate ED, 8-11 moderate ED and 5-7 severe ED (10).

### Statistical analysis

Participating patients were divided into two groups: Patients with UI (Group-1) and without UI (Group-2) problems. Partners of the women in the two groups were compared to detect any possible statistically significant differences regarding erectile function. The categorical variables between the groups were compared using the Chi-Square Test, and the continuous variables were compared using the Student t-Test. P<0.05 was considered statistically significant.

## RESULTS

The mean ages of the 30 group-1 and the 30 group-2 patients were, 45.2±6.3 and 42.5±4.4 years, respectively; and, the mean ages of the partners of the group-1 and group-2 patients were, 47.5±4.6 and 45.2±4.6 years, respectively. The difference between the two groups was not statistically significant. BMI values of the participants were calculated as the body weight in kilograms divided by the square of the height in meters. BMI <18.5 was considered thin, 18.5 to 24.0 normal, 25 to 29.9 overweight, and 30 to 39.9 obese (11). Futhermore, 20 patients had urinary leakage during sexual intercourse in group 1 and the husbands of them were informed about their urinary incontinence. Only 6 patients had ED before the onset of urinary incontinence of the partners of group 1. The group-1 values for the duration of the UI, daily pad use, number and type of deliveries, and (UDI-6) and (IIQ-7) scores are presented in Table-1. The demographic characteristics of group-1 and group-2 patients are summarized in Table-2. The mean length of marriage; duration of ED in group-1 and group-2 were 18.4±4.7; 16±4.8 and 3.9±1.1; 3.4±0,13 years, respectively. In group-1, 9 patients (30%) had UUI, 8 (27%) had SUI and 13 (43%) had MUI. Those with UUI and MUI did not have a history of medical treatment and the SUI patients did not have a history of pelvic surgery. None of the partners of the patients in either group had previously consulted a physician for ED. The partners of patients in both groups were asked to complete the IIEF-5. The results for the group-1 showed that 15 (50%) of the partners had mild ED, 11 (36.6%) %) had moderate ED and 1 (3.4%) had severe ED, while 3 (10%) had no complaints associated with ED. Among the partners of the group 2 patients 15 (50%) had mild ED, 7 (23.3%) had moderate ED and 1 (3.4%) had severe ED, and 7 (23.3%) no complaints associated with ED. ED severity in partners of group 1 patients was significantly higher compared to the partners of group 2 patients (p <0.05). The distribution of ED among partners of group 1 and group 2 patients is shown in Table-3. However, a statistically significant difference in the distribution of ED severity between group 1 and group 2 partners or a relationship between the type of UI in the group 1 patients and the severity of the ED in their partners was not demonstrable (p=0.75) (Table-4).

**Table 1:** Demographic characteristics of Group-1 patients.

Age (years)		45.2 ± 6.3
Number of pads used per day		1.9 ± 0.8
Number of patients with ED before the onset of urinary incontinence		6 (%20)
Duration of incontinence (years)		3.2 ± 1.7
Urinary leakage during sexual intercourse		20 (%66.6)
**Type of incontinence**		
	Stress Incontinence (SUI)	8 (27%)
	Urge Incontinence (UUI)	11 (36.5%)
	Mixed Incontinence (MUI)	11 (36.5%)
Number of Births		2.4 ± 0.9
**Delivery type**		
	NSV	16 (53%)
	C/S	14(47%)
UDI-6		11.5 ± 2.8
IIQ-7		13.1 ± 3.3

**NSV** = Normal Spontaneous Vaginal Route; **C/S** = Cesarian Section; **UDI-6** = Urogenital Distress Inventory Scale; **IIQ-7** = Incontinence Impact Questionnaire-7)

**Table 2:** Demographic characteristics of Group-1 and Group-2 patients.

	Group 1 (n=30)	Group 2 (n=30)	p value
Age (years)	45.2±6.3	42.5±4.4	0.06
Partner's Age (years)	47.5±4.6	45.2±4.6	0.06
Partner IIEF-5	18±5.1	21.7±4.5	0.005*
Duration of partners' ED (years)	3.9±1.1	3.4±0.9	0.13
BMI (kg/m2)	25.5±1.5	25.5±1.3	0.85
Number of births	2.4±0.9	2.1±0.7	0.13
Marriage (years)	18.5±5.4	16±4.8	0.15
Postmenopausal	20 (66.6%)	14 (46.6%)	0.12

* p <0.05 is statistically significant; **BMI** = Body mass index; **IIEF-5** = International Erectile Function Index; **ED** = Erectile dysfunction

**Table 3:** ED distribution among the husbands of Group-1 and Group-2 patients.

	Group-1 (n=30)	Group-2 (n=30)	p value
No ED	3 (10%)a	7 (23.3%)	0.48
Mild ED	15 (50%)	15 (50%)	
Moderate ED	11 (36.6%)	7 (23.3%)	
Severe ED	1 (3.4%)	1 (3.4%)	

**ED =** Erectile dysfunction

**Table 4:** Relationship of UI to ED severity.

		Type of Incontinence			
		Urge	Stress	Mixed	P value
IIEF stage	Advanced ED	1 100.0%	0 0%	0 0%	
	Moderate ED	3 27.3%	2 18.2%	6 54.5%	
	Mild ED	4 26.7%	5 33.3%	6 40.0%	0.75
	No ED	1 33.3%	1 33.3%	1 33.3%	
**Total**		9 30.0%	8 26.7%	13 43.3%	

## DISCUSSION

Urinary incontinence (IU) is a serious health problem, with adverse effects on not only the physical, social and economic quality of life of the women but also their sexual lives (3). Its prevalence varies between 15 % and 52%. The wide difference in these estimations was due to the differences in the groups studied and the variation in the definitions of UI used in the studies (6, 12). The prevalence of the female UI has been found to be over 50% in Turkey (2). ED affects approximately 5% to 20% of the male population and has adverse effects on the quality of life of the patient and of his partner and family (13). This study has demonstrated that the erectile function in the partners of female patients with complaints of UI is reduced.

Although there are many studies on the adverse effects of UI on the sexual lives of female patients (6, 7), there is a paucity of investigations on the erectile functions of their husbands (8, 9). Nilsson et al. have demonstrated that UI lowered the quality of the sexual lives of women that a third of whom had involuntary urinary leakage during sexual intercourse and more than 50% had expressed the anxiety of being repulsive to the other party (7). Sen et al., on the other hand, have shown that sexual function of patients with SUI an UUI was not significantly altered as compared to the controls but that sexual function in MUI patients was significantly lower than in controls. The investigators also have shown that the sexual functions of patients with and without pelvic organ prolapse (POP) had been similarly affected (14). In our study, however, a significant relationship between the type of UI and severity of ED was not observed.

Narin et al. compared the sexual functions of 28 sexually active female patients who had a trans obturator tape (TOT) procedure due to SUI and of their partners before and after the surgical treatment. They have observed significant improvements in the Female Sexual Function Index (FSFI) of the female patients and the IIEF-5 scores of their partners, indicating improvement in the sexual lives of the patients and their partners after TOT due to SUI (15).

Lonnée-Hoffmann et al. evaluated the sexual functions of female patients operated due to POP or SUI and the sexual functions of their partners. The sexual functions of the partners before and one year after the operation showed no change or only a slight improvement (16). A study by Margareta et al., involving 109 female Swedish patients with complaints of UI, sudden urge to micturate and urinary frequency showed that the complaints affected detrimentally the sexual lives of both the patients and their partners (8). Again, a study by Bekker et al., on 81 sexually active patients with UI and 108 patients without UI complaints and their sexual partners demonstrated significantly lower sexual functionality in the group with UI and their partners. In addition, UI was observed to cause a significant reduction in intercourse frequency, degree of satisfaction with the intercourse and erectile function of the male partners (9).

In our study it was shown that the erectile function of the partners of patients with UI was relatively lower than that of the partners of the control group of female patients. Though this reduction was statistically significant as mentioned in Table-3, it was nearly in the same erectile status and erectile function of the partners were not related to the type of UI in the female patients. The retrospective design of our study and the relatively small number of patients involved are the main limitations of this study, indicating need for similar studies to be performed with larger number of patients.

## CONCLUSIONS

Female urinary incontinence (UI) has detrimental effects on sexual lives of the patients and their partners as well. Therefore, the partners of female patients consulting with complaints of UI should also be investigated with respect to sexual functions and if necessary, treatments should also be planned for these individuals.
